# 593. Utility of Microbiologic Testing Obtained via Bronchoalveolar Lavage on Asymptomatic Lung Transplant Recipients: A Quality Improvement Study

**DOI:** 10.1093/ofid/ofab466.791

**Published:** 2021-12-04

**Authors:** William Dillon, Tommy J Parraga Acosta, Andrew J Failla, Julio Corrales, Ramesh Mayur, George J Alangaden

**Affiliations:** Henry Ford Hospital, Detroit, Michigan

## Abstract

**Background:**

The utility of surveillance bronchoscopy (SB) in asymptomatic lung transplant recipients (LTR) is controversial. Guidelines regarding the timing of SB and diagnostic testing varies across centers. Studies evaluating the role of microbiologic testing are lacking. Our transplant institute currently performs SB at week 1, and months 1, 3, 6, 9, 12, and 24 post-transplant. We evaluated if routine microbiologic testing obtained during SB impacted clinical management.

**Methods:**

This observational cohort study was performed at Henry Ford Hospital, Detroit, MI and included all LTR done from August 2014 to August 2019. Clinical and laboratory data was abstracted from the electronic medical record Pre/post-SB. Bronchoscopies performed for new or worsening respiratory symptoms, decline in forced expiratory volume at one second ≥10%, new radiographic abnormalities and follow up bronchoscopies to assess stents or recent acute rejection were excluded. Microbiologic tests assessed are shown in Table 2. Management change was defined as reduction in immunosuppression or prescription of antimicrobials. Rate of change in clinical management based on microbiologic test positivity was the primary outcome. Data were analyzed with descriptive statistics.

**Results:**

449 SB in 107 LTR were evaluated. Median age was 63 years, 68% were male. The average number of SB performed per patient was 4.2 (Table 1). The most common microbiologic tests performed were bacterial (435), mycobacterial (427), and fungal including *Pneumocystis jirovecii* (1022) (Table 2). The rate of test positivity and resultant change in management are shown in Table 3. The rate of test positivity was highest for bacterial (54%), fungal (27%) and viral tests (6%) with management changes in 12%, 2%, and 3% respectively.

Table 1. Patient Demographics

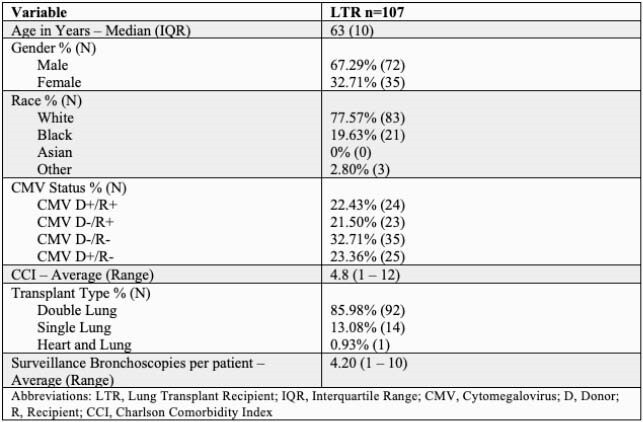

Table 2. Rate of Microbiologic Testing per Surveillance Bronchoscopy

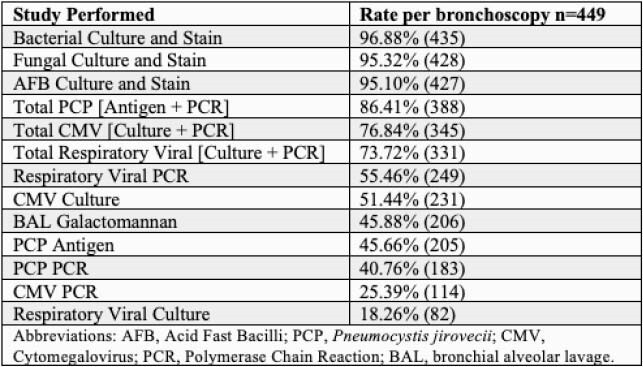

Table 3. Rate of Microbiologic Positivity and Management Change per Surveillance Bronchoscopy

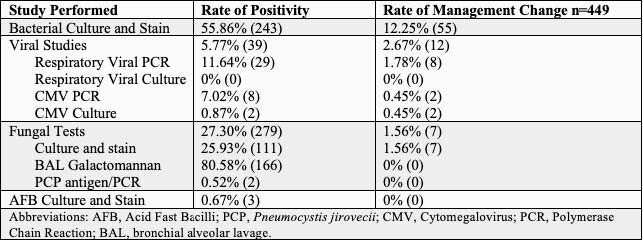

**Conclusion:**

This is the largest study to specifically evaluate the role of routine microbiologic tests during SB in LTR. Bacterial cultures may be appropriate due to higher rates of management changes. However, routine fungal, AFB, and viral studies are unnecessary due to low true positivity, and consequent low rate of management changes. This represents an important opportunity for diagnostic and antimicrobial stewardship.

**Disclosures:**

**All Authors**: No reported disclosures

